# Placebo stimulates neuroplasticity in depression: implications for clinical practice and research

**DOI:** 10.3389/fpsyt.2023.1301143

**Published:** 2024-01-10

**Authors:** Jeremy Seymour, Nigel Mathers

**Affiliations:** ^1^Retired Consultant Psychiatrist, Rotherham Doncaster and South Humber NHS Trust, Rotherham, United Kingdom; ^2^Emeritus Professor, Clinical Medicine, School of Medicine and Population Health, University of Sheffield, Sheffield, United Kingdom

**Keywords:** neuroplasticity, placebo, depression, mechanism of action, fronto-limbic circuits

## Abstract

Neither psychological nor neuroscientific investigations have been able to fully explain the paradox that placebo is designed to be inert in randomized controlled trials (RCTs), yet appears to be effective in evaluations of clinical interventions in all fields of medicine and alternative medicine. This article develops the Neuroplasticity Placebo Theory, which posits that neuroplasticity in fronto-limbic areas is the unifying factor in placebo response (seen in RCTs) and placebo effect (seen in clinical interventions) where it is not intended to be inert. Depression is the disorder that has the highest placebo response of any medical condition and has the greatest potential for understanding how placebos work: recent developments in understanding of the pathophysiology of depression suggest that fronto-limbic areas are sensitized in depression which is associated with a particularly strong placebo phenomenon. An innovative linkage is made between diverse areas of the psychology and the translational psychiatry literature to provide supportive evidence for the Neuroplasticity Placebo Theory. This is underpinned by neuro-radiological evidence of fronto-limbic change in the placebo arm of antidepressant trials. If placebo stimulates neuroplasticity in fronto-limbic areas in conditions other than depression - and results in a partially active treatment in other areas of medicine - there are far reaching consequences for the day-to-day use of placebo in clinical practice, the future design of RCTs in all clinical conditions, and existing unwarranted assertions about the efficacy of antidepressant medications. If fronto-limbic neuroplasticity is the common denominator in designating placebo as a partially active treatment, the terms placebo effect and placebo response should be replaced by the single term “placebo treatment.”

## Introduction

Definitions in placebo are important, and this article initially uses terms agreed by international consensus in 2018 (1). This consensus confirms a paradoxical difference between placebo response and placebo effect. Placebo response applies exclusively to clinical trials, where placebo is used on the basis that it should be as inert as possible and have no clinical consequences, to test the effect size of a defined intervention by determining the difference between the inert placebo and the active treatment.

By contrast, placebo effect refers to clinical interventions which are not intended to be inert: such placebo effects have been used (2), either knowingly or unknowingly, by clinicians to help patients recover since the time of Hippocrates and have a substantial impact on outcomes in clinical care (3), particularly depression (4). There is also a general consensus that it is ethically justifiable to use placebo effects in clinical practice to help patients, provided no subterfuge is involved and the use of placebo is discussed (1, 5).

Placebo response has been studied far more than placebo effect, because of the huge volume of randomized controlled trial (RCT) evidence available from assessment of medical interventions. The quest to find factors (mediators or moderators) that predict placebo response has proved elusive (6).

Depression in particular provides an opportunity to study placebo, since placebo response is known to be high in RCTs of antidepressant treatments, at all levels of depression severity (7, 8). However, depression is a multifaceted condition in which it is difficult to construct pure RCTs to assess treatment outcomes due to difficulties in blinding raters and subjects. In addition, trials of both antidepressant medication and psychological therapy, the two mainstays of treatment, have been subject to accusations of bias because of vested interests (9, 10). This does not detract from the robust finding in the literature that the placebo response is consistently higher in antidepressant trials than in any other medical or psychiatric condition, accounting for approximately 70% of observed improvement in antidepressant trials (11) compared with approximately 50% of observed improvement in all other conditions (12, 13).

Traditional explanations for this difference are that depression is a condition that attracts a high placebo response; or that people who are susceptible to depression are intrinsically likely to be placebo responders; or that there is regression to the mean (14) (regression to the mean arises in one sample of a random variable is extreme, the next sampling of the random variable is likely to be closer to its mean). However, there is little evidence to support any of these explanations, which are laden with value judgments regarding depressed patients.

Neuroplasticity is a topic that has dominated academic publications in translational psychiatry over the last two decades. Neuroplasticity is defined as the ability of the central nervous system to change its activity in response to intrinsic or extrinsic stimuli by reorganizing its structure, functions or connections (15).

In 2016, Rief et al. (16) from the discipline of academic psychology introduced the hypothesis that placebo effect and placebo response trigger neuroplasticity in depression and psychosis, such that placebo is in itself a partially active treatment. This radical hypothesis, if confirmed, has far reaching consequences for interpretation of all clinical trials, particularly those for depression treatment, as well as for the use of placebo to help patients in clinical practice.

This narrative review examines interactions between depression, placebo, and neuroplasticity, and provides updated evidence that placebo itself induces neuroplasticity. The association between neuroplasticity and placebo are referred to in this article as the Neuroplasticity Placebo Theory, and the evidence is drawn from the rapidly developing field of translational psychiatry which lends support to the hypothesis that placebo stimulates neuroplasticity.

This article is in four sections. Firstly, modern thinking on how placebo works is described. Secondly, evidence is examined that neuroplasticity underpins the pathogenesis of dysfunctional fronto-limbic circuits in depression. Thirdly, studies are referenced which provide supportive evidence that stimulating neuroplasticity is a common denominator in all treatment modalities of depression, including psychological therapies. Fourthly, the smaller evidence base that placebo stimulates neuroplasticity is examined. Finally, if it is accepted and established that placebo does indeed induce neuroplasticity, the implications for clinical research and clinical practice are considered in the discussion section.

## Section 1: neuropsychology and neuroscience underpinning placebo

Since placebo was described 70 years ago (17), there have been repeated attempts to apply scientific rigor to its puzzling properties (18). The last 20 years have seen incremental advances in a wide range of disciplines, but have not provided a cogent unifying scientific explanation for the mechanisms underlying placebo, until now.

### Advances in psychological theories of placebo

Academic psychology has evoked learning theory, classical conditioning, and expectancy as the context for verbal, contextual and social cues generating treatment expectancies (19, 20). For example, many of us associate taking medication with feeling better from our childhood experiences, so expectation is created that taking medication in a drug trial will help, even if it is a sugar pill placebo.

Learning theory, conditioning and expectancy have been regarded as competing theories to explain placebo, although Colloca and Miller (20) have suggested amalgamating these ideas into a single integrated learning model. Furthermore, Ashar et al. (21) have developed a sophisticated ‘effective appraisal account’ model of placebo in which the brain incorporates precognitive learnt associations into appraisals of future wellbeing. Thus appraisals shape associative learning, based on what has been learnt from experience. Allying this to neuro-radiological changes in the brain during a placebo condition in mood disorders, pain, and Parkinson’s disease, Ashar et al. (21) found that appraisals reliably engaged the default mode network as centrally important in the placebo condition. The default mode network represents areas that are more active during times of brain quiescence compared to cognitive activity, and usually involves fronto-limbic areas including the left dorsolateral prefrontal cortex (DLPFC), the anterior cingulate cortex and ventral striatum. These are the anatomical areas associated with the brain making appraisals during placebo, and as abnormalities in these areas are also evident in depression, a co-location link between depression and placebo is invoked, potentially explaining why placebo response is so high in trials of antidepressant treatment (22). However, this is an association rather than causation, and while it has been suggested that placebo is a neuromodulator in depression (23), this remains unproven.

The study of Ashar et al. (21) is an important advance in the understanding of placebo from a psychological perspective, but, as the authors acknowledge, it fails to explain why placebos persist and do not naturally extinguish (24). A remarkable feature of both placebo effect and placebo response is that they persists for several weeks (24, 25). A limitation of all psychological theories of placebo is therefore that there is no ready explanation for persistence, unless an additional process such as neuroplasticity is invoked.

### Advances in neurochemical and neuroscientific theories of placebo

Advances in the psychological understanding of placebo have been paralleled by increasing knowledge of the neuroscientific basis of placebo. Molecular and genetic contributions to placebo have been delineated, for example, through the reproducible neurochemical changes in dopamine levels, monoamines and opioids (26), demonstrated in Parkinson’s disease (27), mood disorders (28) and pain states (29). As in psychological theories of placebo, the same neuroanatomical brain regions are involved, principally the default mode network. It is possible that the default mode network is sensitized to respond to placebo influences in a different manner in CNS disorders.

The focus on neurochemical changes in Parkinson’s disease, mood disorders and pain states has its limitations, as it is not clear whether the results are transferrable to non CNS disorders, for example, placebo influences in asthma or dermatitis.

Readers interested in the neuroscience underpinning placebo are referred to the reviews by Cai and He (30), and Wager and Atlas (31), but it can be concluded that solely neuroscientific studies cannot completely answer the question: “how can placebo effect and placebo response be explained in diverse medical disorders that are outwith the CNS?”

### Summary

While some authors have sought to explain placebo in purely psychological terms (7, 20), or in purely neuroscientific terms (32), most literature reflects a general consensus that psychological and neuroscientific explanations are complementary and of equal importance. Explaining placebo requires contributions from diverse areas of literature (20, 33, 34), but it is only by invoking an additional process such as neuroplasticity that creates the potential to bridge the gap between psychological and neuroscientific explanations of placebo.

## Section 2: noxious humoral stimuli in depression trigger dysfunctional neuroplasticity

The monoamine hypothesis of depression was first articulated in 1965 (35) and suggested that systemically secreted hormones induced by stress interacted with and induced change in brain neurochemicals, principally monoamines, via the Hypothalamic–Pituitary–Adrenal (HPA) axis. Tricyclic antidepressants and monoamine oxidase inhibitors, which were discovered by serendipity, were thought to exert their mode of action by correcting this chemical imbalance. With the advent of functional Magnetic Resonance Imaging (fMRI) and Positron Emission Topography (PET) it became clear that depression at the more severe end of the spectrum is a disorder of structure as well as function, with marked abnormalities demonstrable in fronto-limbic circuits (36) that are reversible with treatment (37), i.e., they are not just epiphenomena.

Neuroplasticity has been unequivocally demonstrated to be disrupted in mood disorders and animal models of stress (38). Chronic stress precipitates and exacerbates depression via neuroplasticity, but more importantly antidepressant treatments (in the broadest sense) stimulate opposing effects to enhance neuroplasticity and reverse the changes induced by stress. While the exact role of neuroplasticity in the genesis and management of depression (and other overlapping disorders such as anxiety and psychosis) has yet to be elucidated, neuroplastic change demonstrably effects both structure and function in human and animal models of depression (15).

The monoamine hypothesis has therefore been superseded by the formulation that depression is a disorder of brain neuroplasticity (15, 39), probably triggered by over-activity of the HPA axis.

Furthermore, if abnormal fronto-limbic circuits specific to depression have formed, the aim of all antidepressant treatment is firstly to disrupt the abnormal circuits and then to promote their replacement with “normal” circuitry, via a process of neuroplasticity (40). This model complements psychological theories on the genesis and management of depression (41).

Neuroplasticity has now been described in structural terms, with direct evidence of stimulation of new dendritic spine growth and interconnections which can be observed *in vivo* (42); in functional terms, through stimulation of new synaptic morphology equivalent to “upregulation of receptors” (43); and in biochemical terms through description of cellular mechanisms. The biochemical link is particularly important, as blockade of the N-methyl-D-Aspartate receptor on glutamate neurons stimulates release of Brain Derived Neurotrophic Factor (44), which increases synaptogenesis and dendritogenesis (42). There is also some evidence that blood BDNF levels-as a marker for neuroplastic activity-are correlated with antidepressant response (45).

In summary, it has been known since the last century that neurochemical explanations of both the pathogenesis and management of depression do not explain the whole picture, and links between monoamine abnormalities and neuroplasticity are increasingly evident (46). The conclusion of more than 20 years of translational and clinical research is that adverse neuroplasticity is centrally involved in the pathogenesis of depression, resulting in aberrant resting state functional connectivity in fronto-limbic circuits subserving emotion, reward processing, and executive functioning. This approach is consistent with psychological theories of depression such that neuroplasticity and psychological theories can now be integrated (41). The corollary is that stimulating neuroplasticity is also now a prime target for all antidepressant treatment interventions (40): this has been elegantly summarized in the reviews by Pittenger and Duman (38) and Duman and Price (41).

## Section 3: neuroplasticity is a universal brain process that is fundamental to all antidepressant treatment

The explosion of translational research into neuroplasticity, and the ability to track it through neuro-radiological techniques (47), has clarified the central role of neuroplasticity in both neurodevelopment and central nervous system (CNS) repair. At an early stage of postnatal human brain development, glutamate and gamma-aminobutyric acid (GABA) are the only neurochemicals identifiable in the CNS: glutamate and GABA are therefore centrally involved in stimulating physiological neuroplasticity (48). This applies throughout the lifespan, with neuroplasticity playing a central role in maintenance of brain function throughout. Some fronto-limbic brain regions are more susceptible to neuroplastic change than other brain regions, for example the hippocampus is particularly sensitive (49), and can even generate new cells in response to stimuli (neurogenesis), as well as the dendritogenesis and synaptogenesis that are the core of neuroplasticity (42).

Diverse stimuli initiate neuroplasticity in different brain regions over different time frames. Musical training induces neuroplasticity in the dorsal auditory stream region (50). Playing the computer game Super Mario induces neuroplasticity in the right hippocampus, right DLPFC, and bilaterally in the cerebellum (51). Yoga induces gray matter volume change in the left insular, frontal operculum and orbitofrontal cortex (52). Any drug that crosses the blood brain barrier, prescribed or recreational, exerts part of its effect by interacting with receptors and stimulating neuroplasticity (53): it is now routine to be able to track neuroplastic changes in drug development *in vivo* using sophisticated neuroimaging (37, 42, 54). The therapeutic potential of neuroplasticity in many fields of medicine, but particularly psychiatry, has yet to be realized (40, 55).

Depression represents a special case of neuroplasticity for two reasons. Firstly, adverse neuroplastic change has already occurred in the brain in the genesis of depression, with formation of the abnormal circuits demonstrable on fMRI. Secondly, the relevant brain areas in depression are interconnecting pathways between the DLPFC, the limbic system, and the hypothalamus. These pathways are particularly sensitive and susceptible to neuroplastic change. There is now strong evidence that neuroplasticity is centrally involved in the therapeutic action of diverse antidepressant treatment modalities, including electroconvulsive therapy (56, 57), psychological therapy (58), exercise (59), and medication (15, 39, 54).

Much has also been learnt about neuroplasticity in depression from investigating the mode of action of ketamine (39, 54). Ketamine was discovered by serendipity, and the original description noted rapid improvement in depressive symptoms within 45 min in depressed patients coincidentally receiving ketamine as an anesthetic (60). Ketamine is unusual as an anesthetic in exerting its mode of action by interrupting association pathways between the thalamo-cortical and limbic systems to induce unconsciousness (61)—most other anesthetics work on the reticular activating system—and this anatomical location of site of action is relevant to its antidepressant effects. The speed of antidepressant action of ketamine has revealed two types of neuroplasticity: ionotropic, which acts within hours, and metabotropic, acting over weeks (62).

The process of improving clinical outcomes in depression by managing neuroplasticity (15, 39, 54), is now the predominant research avenue for developing novel antidepressant treatments.

## Section 4: evidence that placebo stimulates neuroplasticity

Rief et al. (16) first postulated that placebo stimulates neuroplasticity in depression and schizophrenia, based on a decade of psychology research into the placebo response in mental illness, but their hypothesis was rooted in psychological expectation theories of placebo in depression without reviewing the wider context (63). Their observation that placebo stimulates neuroplasticity was from the perspective that treatment context affects psychopharmacological interventions, and, for example, the prescription of antidepressant medication should be accompanied by exercise.

Their hypothesis has subsequently been supported by data from the Establishing Moderators and Biosignatures of Antidepressant response for clinical Care (EMBARC) series of studies, which set out to investigate clinical moderators and biological moderators and mediators of antidepressant response (64). Specifically, the studies compared prospectively a range of markers, including fMRI and cerebral blood perfusion, in an adequately powered trial of patients with Major Depressive Disorder (MDD) who received either sertraline or placebo over an 8-week period. A striking and unexpected finding, not anticipated in the original aims and objectives of the study, was the high response rate of the placebo group, resulting in a negligible effect size for sertraline treatment. Overall, 33% of subjects randomized to the placebo group achieved remission compared to 37% of the active sertraline treated group (65, 66) strongly suggesting that placebo is a partially active treatment in MDD.

The second striking finding of the EMBARC studies was that the group receiving placebo demonstrated cerebral perfusion and functional neuro-radiological change suggestive of neuroplasticity in fronto-limbic areas, albeit in slightly different brain regions to the group receiving sertraline. This unexpected finding prompted some of the EMBARC study group to conduct a systematic review (67) that sought functional neuroimaging correlates of placebo response in subjects with anxiety/depressive disorders. The 12 extracted studies for depression found that in patients where placebo induced antidepressant improvement occurred, this correlated broadly with abnormalities in the default mode network, known to mediate depression (36), with prominent additional activity in the ventral striatum, rostral anterior cingulate cortex, orbitofrontal cortex and particularly in the DLFPC. These brain areas show abnormal activity in depression, so it is a significant finding that similar abnormalities are seen with placebo.

Overall, the findings of the EMBARC series of studies lend support to the hypothesis that placebo stimulates neuroplasticity, a serendipitous finding given the original aims and objectives of the study (64).

## Discussion

As with many discoveries in depression, serendipity has played a prominent role. The EMBARC studies provide coincidental data that placebo induces fronto-limbic stimulation of neuroplasticity in MDD patients, lending indirect support to the hypothesis of Rief et al. (16) that placebo stimulates neuroplasticity.

**Figure 1:**
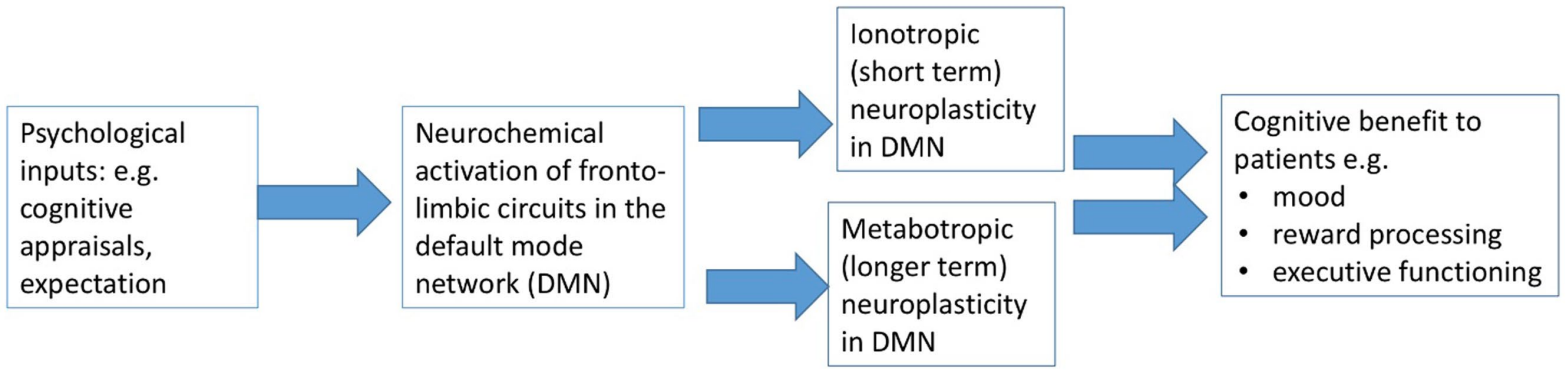
The Neuroplasticity Placebo Theory in CNS and non-CNS disorders.

Synthesizing the evidence from the four sections above, the Neuroplasticity Placebo Theory states that placebo effect and placebo response are equivalent, and are active interventions associated with neuroplasticity. The link between psychological and neuroscientific explanations of placebo is that expectation triggers neuroplasticity in fronto-limbic areas that subserve mood, executive functioning and emotion (see Figure 1).

Neuroplasticity is the common denominator, exerting similar measurable neurobiological activity in fronto-limbic areas of the brain in the different settings of clinical practice and clinical trials. While placebo is active in almost all clinical trials across every medical intervention, it is particularly prominent in trials of depression as fronto-limbic areas are already sensitized by the process of developing depression. The Neuroplasticity Placebo Theory is able to explain the paradox that placebo effect and placebo response apparently differ, and posits that placebo *effect* and placebo *response* are terms that should become redundant, to be replaced by the single term placebo *treatment.*

There is therefore sufficient evidence from the EMBARC studies and the systematic review of Huneke et al. (67) to conclude that placebo is a partially active treatment in depression through stimulation of neuroplasticity. This is the first article to suggest that neuroplasticity is generalizable to all placebo influence, not just depression, and to review evidence that placebo stimulates neuroplasticity.

The Neuroplasticity Placebo Theory potentially has far reaching implications for research and clinical practice.

Firstly, clinicians are using placebo treatment in many interactions with their patients, an intervention that is changing their patients’ brain morphology, so clinicians should explain this to patients in the context of the principles of informed consent (1, 5), as with any other treatment intervention. It is unclear if such an explanation to patients would dissipate the benefits of placebo.

Secondly, the Neuroplasticity Placebo Theory suggests that in general all existing RCTs are variably contaminated by bias as placebo response varies with trial conditions. The results of RCTs are not invalidated by this observation because of the power of randomization (14), but should be interpreted with caution. If clinical triallists wish to design RCTs that minimize the placebo treatment influence—and thus provide a better assessment of the effect size—interventions could be delivered remotely rather than via human contact with a research assistant or clinician, and subjects with comorbid depression could be excluded from clinical trials. Thirdly, it is known that placebo treatment persists (24, 25), but it is not known for how long it persists. Future trial design should incorporate longer term follow up of outcomes in order to better determine effect size, and should consider adding mixed methods research (68, 69) to the evaluation of short and long term outcomes.

Finally, specifically for depression trials, depression as a disorder is unique in that there is a relatively large impact on the control arm of RCTs, which may undermine conclusions regarding effect size. Several authors have evoked the small effect size in trials of antidepressant medications to repeatedly assert (7, 9, 70, 71) that as antidepressants have limited efficacy vs. placebo, they should be replaced by psychological therapy and exercise to treat depression at all levels of severity, and thus avoid the side effects of antidepressants. These opinions are controversial (72), and have generated a disproportionate degree of attention in media and social media (73), and contribute to the potentially harmful decision by many patients with MDD worldwide to refuse antidepressants (74).

The Neuroplasticity Placebo Theory helps to resolve this controversy by clarifying that those authors, who have based their assertions on the outdated concept that depression is caused by a chemical imbalance (70, 71), are asking the wrong question: instead of asking “Why is the effect size so small?,” the correct question is “Why is the placebo response so high?,” to which the answer is “Neuroplasticity.” It can be concluded that the work of Kirsch and Moncrieff has been a major contribution to the literature in drawing the attention of patients, clinicians and commissioners to the importance of psychological therapy and exercise in a stepped model of care (75). However, the sophisticated statistical analysis by Cipriani et al. (76, 77) is more relevant in deciding the place of antidepressants in the management of MDD, since the observations of Kirsch and Moncrieffe on effect size in depression of all grades of severity have been superseded by advances in knowledge of placebo and neuroplasticity (39, 41, 67).

If placebo is a partially active treatment, its place in the management of depression could be tested further by a RCT design for depressed subjects that compares placebo (i.e., a sugar pill in a drug trial setting) with no treatment, if such a trial could be deemed ethically justifiable (78). The persistence of placebo response (24, 25) is supportive evidence for the Neuroplasticity Placebo Theory, which invokes metabotropic neuroplasticity as the explanation for persistence. However, further research is also required into how long the benefits of placebo treatment persist in other medical interventions. More prospective research is needed with long term follow up to clarify if exercise or counseling, which also act on fronto-limbic areas, confer any synergistic benefit to outcomes when combined with other neuroplasticity-inducing antidepressant treatments (40).

This article is a narrative review rather than a critical review, a systematic review, or a meta-analysis. It presents no new data in support of the Neuroplasticity Placebo Theory. As such it is aimed at practicing clinicians and has sought support for the theory by synthesizing ideas and evidence from diverse sources of literature; in particular the novelty of linking the hypothesis of Rief et al. (16) with outcome data from the EMBARC series of studies (65). This underlines the importance of integrating psychological and neuroscientific formulations, in research as well as clinical practice, to best help patients.

## Data availability statement

The original contributions presented in the study are included in the article/supplementary material, further inquiries can be directed to the corresponding authors.

## Author contributions

JS: Writing – original draft. NM: Writing – review & editing.
